# The Dignity and Importance of Dentistry

**Published:** 1922-04

**Authors:** Edward C. Kirk


### THE DIGNITY AND IMPORTANCE OF DENTISTRY

BY EDWARD C. KIRK, D.D.S. SC.D.

An emiment educator in an address on "Correlation in the Teaching of Dentistry and Medicine," quotes Mr. Abraham Flexner (see *Dental Cosmos,* page 242), Secretary of the General Education Board, as saying: "We have come to see in the last few years that dentistry is a branch of medicine of the same dignity and importance as pediatrics or any other specialty. The new school of medicine will, it is hoped, undertake to place training in dentistry on the same academic and scientific level as medicine and surgery."

Taken at its face value the above-quoted statement is an interesting demonstration of medical psychology that either knowingly or subconsciously portrays the inner workings of the medical professional mind, as that mind is prone to react toward dental professional stimuli. A brief critical analysis of the statement may therefore be not without interest and may possibly lead to some conclusions useful in bringing about such a correlation of teaching in dentistry and medicine as will redound to the good of all concerned.

First then, "We have come to see in the last few years that dentistry is a branch of medicine of the same dignity and importance as * * * any other specialty."

It is fair to assume that "We" in this case means the medical profession or at least those competent to speak with authority on behalf of the medical profession.

It is now over eighty years since Chapin A. Harris and Horace H. Hayden, both medical men, imbued with precisely the same idea as we have quoted from Mr. Abraham Flexner, made overtures to the Trustees and Faculty of the Medical School of the University of Maryland with a view to placing "training in dentistry on the same academic and scientific level as training in medicine and surgery." History records that their proposal was rejected with scorn, and with the result that dentistry, now hailed as a dignified and important specialty of medicine, on being refused admission to the medical household established its own centers of education, developed its own literature and professional associations, and an autonomous control of its professional activities that thus far have enabled it to survive and work out its own destiny. In short, dentistry has justified its right to survive despite the fact that it has been treated by the medical schools and their progeny with the scant courtesy usually accorded to an illegitimate child through more than three-quarters of a century of its existence,—denied recognition for its attainments, refused admission to medical associations and councils, scoffed at as a mechanic art and the status of its degree sneered at as the impertinent badge of a partial culture.

Now we seem to have been discovered—not only discovered, but we are found to be a profession of dignity and importance equal in these desirable characteristics to the other recognized specialties of medicine. Now what is to happen? Pursuing the psycho-analysis of our quoted text the answer is not far to seek. *Voila!* "The new school of medicine will, it is hoped, undertake to place training in dentistry on the same academic and scientific level as training in medicine and surgery." That is to say, now that we are discovered and found to be dignified and important the *medical school* will undertake to place training in dentistry on the same academic level as training in medicine and surgery.

We realize of course that our quotation is from an address in which this hopeful suggestion was made with reference to a particular medical school and under the especial circumstances of a consideration of its academic organization, and is therefore subject to the qualification of that limitation. Nevertheless, the statement bears the ear-marks of that ancient prophetic axiom that out of the fullness of the heart the mouth speaketh.

Then what? There is no intimation as to the future relative status of dentistry to medicine after the medical school has undertaken to reorganize the basis of education in the dental school, so we infer that the utmost that can happen will be that the medical school and its progeny will continue to realize that the dental school and its progeny are dignified and important.

But will that proposed rearrangement of affairs be a material benefit either to dentistry or medicine, Let us consider for a moment the circumstances surrounding this new medical discovery of the dignity and importance of dentistry. History records that after nearly three-quarters of a century of consignment to medical oblivion dentistry suddenly loomed large on the horizon of medical consciousness through the clinical findings of Sir William Hunter with respect to oral sepsis and its relation to systemic disease.

In many respects the publication of Hunter's papers was the most important contribution ever made to either dental or medical literature. For dentistry they acted as a potent counter-irritant that compelled a revision of dental technique in harmony with the vital activities of the oral tissues and the pathogenic flora of the mouth cavity. To medicine they opened the door to therapeutic possibilities before unknown or neglected, and compelled a study of conditions that had been tacitly regarded as unimportant, possibly the mechanical and therefore professionally undignified art of the dentist. Hunter's communications threw a new light on the matter, a new light literally, for in principle the relation of mouth infection to systemic disease was a part of the literature of dentistry worked out to a demonstration by W. D. Miller, a dentist almost a quarter of a century before Hunter's studies were published. So it will be readily seen how it was that the dignity and importance of dentistry came to be discovered and is not a thing of recent development, for it existed long before Mr. Abraham Flexner or the medical profession found it out.

This leads us to the conclusion that the mere fact of the belated discovery of the dignity and importance of dentistry by the medical profession ought not in the nature of the case to confer the right upon the medical school to undertake "to place training in dentistry on the same academic and scientific plane as training in medicine and surgery" or do any other thing that will eventually place dental education under medical control. The reason is obvious. Some years ago the now deceased editor of a Philadelphia local newspaper, as the Christmas season was approaching, cautioned his readers somewhat as follows: "As the joyous Christmastide approaches do not make the unfortunate mistake of trying to buy your plum pudding at the plumbers." Consistency, as well as the public interest, not to speak of the interest of dentistry, should determine that dental education is a function of the dental profession and that dental educators are better qualified to produce educated dentists than medical educators are.

The assumption that the medical profession has or should have monopolistic control of those biologic sciences that are the foundations of the healing art is a fallacy that dies hard in the medical mind, notwithstanding that the historic pathway of organized medicine is strewn with the wrecks of practical attempts to prove the title of organized medicine to all medical knowledge.

Dentistry has for its whole history been gradually including all those elements of scientific medical knowledge needful for its growth and successful practice. Its academic preparation, its scientific basis is already close to if not on a parity with that required for medical and surgical training. While the dignity and importance of dentistry is a recent medical discovery, dentistry itself can scarcely be said to have yet revealed itself to the medical mind. Some day perhaps a newer type of medicine may take over the control of dental education, but—"not yet, Amarillo, not yet!"

